# Glucose Transporter Expression in an Avian Nectarivore: The Ruby-Throated Hummingbird (*Archilochus colubris*)

**DOI:** 10.1371/journal.pone.0077003

**Published:** 2013-10-14

**Authors:** Kenneth C. Welch, Amina Allalou, Prateek Sehgal, Jason Cheng, Aarthi Ashok

**Affiliations:** Department of Biological Sciences, University of Toronto Scarborough, Toronto, Ontario, Canada; Tohoku University, Japan

## Abstract

Glucose transporter (GLUT) proteins play a key role in the transport of monosaccharides across cellular membranes, and thus, blood sugar regulation and tissue metabolism. Patterns of GLUT expression, including the insulin-responsive GLUT4, have been well characterized in mammals. However, relatively little is known about patterns of GLUT expression in birds with existing data limited to the granivorous or herbivorous chicken, duck and sparrow. The smallest avian taxa, hummingbirds, exhibit some of the highest fasted and fed blood glucose levels and display an unusual ability to switch rapidly and completely between endogenous fat and exogenous sugar to fuel energetically expensive hovering flight. Despite this, nothing is known about the GLUT transporters that enable observed rapid rates of carbohydrate flux. We examined GLUT (GLUT1, 2, 3, & 4) expression in pectoralis, leg muscle, heart, liver, kidney, intestine and brain from both zebra finches (*Taeniopygia guttata*) and ruby-throated hummingbirds (*Archilochus colubris*). mRNA expression of all four transporters was probed using reverse-transcription PCR (RT-PCR). In addition, GLUT1 and 4 protein expression were assayed by western blot and immunostaining. Patterns of RNA and protein expression of GLUT1-3 in both species agree closely with published reports from other birds and mammals. As in other birds, and unlike in mammals, we did not detect GLUT4. A lack of GLUT4 correlates with hyperglycemia and an uncoupling of exercise intensity and relative oxidation of carbohydrates in hummingbirds. The function of GLUTs present in hummingbird muscle tissue (e.g. GLUT1 and 3) remain undescribed. Thus, further work is necessary to determine if high capillary density, and thus surface area across which cellular-mediated transport of sugars into active tissues (e.g. muscle) occurs, rather than taxon-specific differences in GLUT density or kinetics, can account for observed rapid rates of sugar flux into these tissues.

## Introduction

While hovering at flowers, feeding on sugar-rich nectar, hummingbirds sustain some of the highest aerobic metabolic rates observed among vertebrates [1]. Remarkably, hummingbirds can fuel energetically expensive hovering flight with either endogenous lipids when fasted or with recently ingested sugars when foraging, completely switching between fuel sources over the course of only 30–40 minutes [2]–[4]. Tracking of carbon from ingested nectars with distinct isotopic signatures in expired CO_2_ indicates extremely rapid turnover within the pool of actively metabolized substrates [2], [3]. This rapid flux of sugar from floral nectar to working flight muscles in hummingbirds (and other nectarivorous vertebrates) where it is oxidized, termed the ‘sugar oxidation cascade’ [5], involves the concerted upregulation of sugar transport through the cardiovascular system and across multiple tissue barriers (e.g. intestinal wall, capillary endothelial and muscle fiber membranes). Capacities for the assimilation and absorption of sugar in the gut are enhanced by very high maximal rates of sucrase activity in the hummingbird intestine [6], and high rates of both active and passive sugar movement across the intestinal wall [7], [8]. However, mechanisms governing the flux of sugars from the cardiovascular system to metabolically active tissues is poorly understood in birds generally, and hummingbirds in particular.

The transport of hydrophilic sugar molecules across cell membranes occurs through one of several related facultative transporters comprising the GLUT family of proteins. While 14 members of the GLUT transporter family have thus far been described in mammals [9], the first four to be described, GLUT proteins 1–4 (class I), remain the best characterized [10]. GLUT1 is expressed in almost every tissue in mammals and is thought to provide basal levels of glucose transport [10], [11]. While GLUT3 mRNA is found in most mammalian tissues, protein expression is generally limited to the brain, testes, and skeletal muscle [12], [13]. GLUT2 and 4 play key roles in the hormonal and activity-induced regulation of blood sugar level and uptake rate into tissues [11], [14], [15]. GLUT2 is a key element of the peripheral glucose sensing system [10], [15]. Its expression in mammals is generally limited to tissues with important roles in whole organism energy homeostasis, such as the liver, kidneys, pancreas and intestine [10], [11]. In mammals, increased binding of glucose to GLUT2 following a rise in blood sugar activates pathways promoting insulin release by pancreatic β-cells [10]. GLUT4 expression is limited to skeletal muscle, heart, and adipose tissues [10], [16]. GLUT4 in muscle, heart, and adipose is translocated from intracellular vesicles to the plasma membrane in response to insulin [11] and, through an independent pathway in muscle, increased contractile activity [17], [18], resulting in a dramatic enhancement of glucose uptake.

Blood glucose levels in most mammals are typically below 10 mM and are tightly regulated by regulatory pathways including the insulin/glucagon axis, involving members of the GLUT family of transporters [15], [19]. In contrast, birds exhibit relative hyperglycemia, with blood glucose levels averaging 2–3 times the level seen in comparably sized mammals [15], [19]. In addition, birds appear insulin insensitive, with blood glucose levels either unaffected by exposure to insulin [20], [21] or with moderate hypoglycemia induced only by exposure to supra-physiological insulin levels [22]–[24].

Blood glucose levels in fasted hummingbirds (14±0.4 mM; fasting duration: 1.3 hrs - overnight) are among the highest reported and are considerably higher than levels measured in terrestrial mammals following feeding [19], [25]. Foraging hummingbirds exhibit blood glucose concentrations that are several times higher still (28 to 41 mM) [25]. Hummingbird blood glucose levels remain high when foraging regularly (feeding on nectar every several minutes).

The fact that hummingbirds can rely exclusively on recently ingested sugars to fuel energetically expensive hovering flight implies that the integrated rate of sugar transport from circulation into metabolically active muscle tissue is quite high. Much of the control over rates of glucose uptake and use by metabolically active tissues such as exercising muscle is believed to occur in steps defining the transport of glucose from circulation into these tissues [26]–[31]. In addition, rapid turnover of ingested sugars in the pool of actively metabolized substrates combined with sugar intake rates which significantly exceed immediate catabolic requirements implies sugars can be removed from circulation to build fat stores in the liver and adipose tissue at high rates as well. The fact that even fasted hummingbirds exhibit relatively high blood glucose levels suggests that there may be important differences in the expression or control of one or more members of the GLUT transporter family involved in blood sugar regulation. Additionally, assuming GLUT transporters represent a key rate limiting step in the ‘sugar oxidation cascade’ pathway it is reasonable to hypothesize that there is functional enhancement of GLUT transporter capacity in the vasculature, muscle, liver, adipose and other metabolically active tissues in hummingbirds.

The goal of this study was to characterize expression patterns of class I glucose transporters in the tissues of hummingbirds. Transporter expression was first examined in zebra finches, because better characterization of this species’ genome facilitated primer design and antibody cross-reactivity screening prior to initiating work in hummingbirds. We hypothesized that, similar to other avian taxa, GLUT4 is not expressed in the tissues of either species. Expression of GLUTs was assessed in several tissues using reverse transcriptase (RT) PCR, immunoblotting, and immunohistochemistry.

## Materials and Methods

### Ethics Statement

This study was carried out in strict accordance with the recommendations in the Canadian Council on Animal Care’s Guide to the Care and Use of Experimental Animals. All animal protocols (# 20008396, 20008929, 20009520) were approved by the University of Toronto Laboratory Animal Care Committee.

### Animals

Ruby-throated hummingbirds (*Archilochus colubris*) were captured on the University of Toronto Scarborough (Toronto, Ontario, Canada) campus using modified box traps between May, 2010 and June, 2012 and were sampled between May, 2011 and June, 2012. Zebra finches (*Taeniopygia guttata*) were purchased from local suppliers in January, 2011 and January, 2012 and sampled between February and June, 2011 and January and February, 2012, respectively. All animals were housed in the University of Toronto Scarborough vivarium under standard conditions. Birds were provided food (hummingbirds: Nektar Plus, Guenter Enderle, Tarpon Springs, FL, USA; finches: standard commercial finch seed and millet) *ad libitum* up to the time of sacrifice and all birds were thus considered well-fed immediately prior to sampling. Additionally, tissue from healthy laboratory mice (FVB background strain; 010541-UCD, Mutant Mouse Regional Resource Center, National Institutes of Health, Bethesda, Maryland, USA) was obtained from individuals sacrificed as part of other studies in the University Of Toronto Scarborough Department Of Biological Sciences and included in our study as positive controls. Birds were first anesthetized by exposure to >3% isoflurane, delivered by precision vaporizer, and then euthanized by asphyxiation under nitrogen. Tissues were immediately extracted.

### Reverse Transcription-Polymerase Chain Reaction (RT-PCR)

Primers for D-glyceraldehyde 3-phosphate dehydrogenase (GAPDH) RNA were used to amplify products that served as a positive control. GAPDH primers were designed based on published sequences from chicken [32]. The design of primers for GLUT1, GLUT2, and GLUT3 were based on the putative zebra finch genomic sequence listed in GenBank (Accession # XM_002192008, #XM_002193773.1, and #XM_002190755, respectively). No sequence with sufficient homology to mammalian GLUT4 is published for the zebra finch or any hummingbird species within GenBank. As no consensus sequence for any avian species was available, we adopted the use of a primer set based on the rat GLUT4 sequence, as employed by Sweazea and Braun [33]. We also developed a second, non-overlapping set of primers based on the published sequence in mouse (Accession # NM_009204). The specificity of all primers was examined and verified using BLAST (http://blast.ncbi.nlm.gov/Blast.cgi). All oligonucleotide primers were synthesized by Life Technologies Inc. (Burlington, ON, Canada). Primer sequences are listed in Table 1.

**Table 1:** Oligonucleotide sequences used for Reverse transcriptase PCR.

Primer	Forward sequence	Reverse sequence	Fragment Size	Source
GLUT1	5′ - GCATGATCGGCTCCTTCTCTGT - 3′	5′ - AGCAGCGGCCAGAGAGAGTCGT - 3′	340 bp	GenBank (XM_002192008)
GLUT2	5′ - TTCGCCGTCGGTGGCATGGT - 3′	5′ - CGTGACTGCTCTCCCGGAGATG - 3′	305 bp	GenBank (XM_002193773.1)
GLUT3	5′ - CTTTGTGGCCCTTTTTGAGA - 3′	5′ - ATCTCCACCATGGGGTTCTT - 3′	543 bp	GenBank (XM_002190755)
musGLUT4	5′ - TTCACGTTGGTCTCGGTGCT - 3′	5′ - CGTCGGAAGGCAGCTGAGAT- 3′	449 bp*	GenBank (NM_009204)
GAPDH	5′ - ACGCCATCACTATCTTCCAG - 3′	5′ - CAGCCTTCACTACCCTCTTG - 3′	585 bp	Croissant et al., 2000

* Expected fragment size based on mouse sequence.

Except where otherwise noted, predicted fragment sizes are based on putative zebra finch sequences.

In zebra finches, total RNA was isolated from the pectoralis (P), brain (B), heart (H), liver (L), ankle-extensor muscle group (G; e.g. gastrocnemius, soleus), wrist-extensor muscle group (E; e.g. extensor digitorum longus), kidney (K), intestine (I), and pancreas (A) using the RNeasy Mini Kit (Qiagen, Toronto, ON, Canada) following the manufacturer’s standard protocol. RNA was isolated from hummingbird tissues following the same protocol. However, because tissues masses were so small, wrist-extensor muscle, kidney, and intestine samples from 2 or more individual hummingbirds had to be pooled prior to analysis in some cases. This reduced the effective sample sizes for these tissues and meant that RT-PCR products from all tissues from one individual could not be run on the same gels in some cases. Additionally, unlike in zebra finches, the pancreas was not sampled in hummingbirds due to insufficient tissue mass. RNA was isolated from mouse cardiac tissue and analyzed to detect GLUT4 expression, serving as a positive control.

Tissue from 3–7 individual hummingbirds and 3–5 zebra finches were sampled for use with each primer set, except for the following tissues where, due to limited tissue mass and competing need of tissue for protein expression analysis (see below), samples from 2 individuals were included: GLUT1– hummingbird wrist-extensor muscles (E – pooled from 4 individuals) and zebra finch pancreas (A). GLUT2– hummingbird brain (B), ankle-extensor muscle group (G), kidney (K), intestine (I), and wrist-extensor muscles (E – pooled from 4 individuals) and zebra finch pancreas (A). GLUT3– hummingbird wrist-extensor muscles (E – pooled from 4 individuals) and zebra finch pancreas (A). GLUT4– zebra finch ankle-extensor muscle group (G) and pancreas (A).

Reverse transcription and amplification of cDNA was carried out using the OneStep RT-PCR kit (Qiagen) following the manufacturer’s standard protocol. Each 25 µl reaction mix consisted of 5 µl 5×OneStep RT-PCR buffer, 0.4 mM of dNTP nucleotides, 1 µl of OneStep RT-PCR enzyme mix (containing HotStarTaq DNA polymerase with Omniscript and Senscript reverse transcriptases), and 0.6 µM each of the forward and reverse primers. RT-PCR reactions were carried out in either a DNA Engine® Peltier Thermal Cycler (model PTC0200, Bio-Rad Laboratories Ltd., Mississauga, Ontario, Canada) or in an MJ Research PTC-200 Gradient Thermal Cycler (MJ Research Inc., St. Bruno, Quebec, Canada). A thermal cycle profile was run for 40 cycles including denaturation at 94°C for 1 min, annealing at a primer-specific temperature for 1 min, and extension at 72°C for 1 min. Annealing temperatures were: GAPDH 55°C, GLUT1 65°C, GLUT2 56°C, GLUT3 56.5°C, GLUT4 56°C. Predicted product sizes, based on the putative zebra finch sequence were: GAPDH 585 bp, GLUT1 340 bp, GLUT2 305 bp, GLUT3 543 bp, GLUT4 449 bp (based on mouse sequence). Negative control reactions, conducted as described above except for the omission of RNA, were conducted using all primer sets. Reaction products were analyzed on 1.5% agarose gels along with a 100 bp DNA ladder (GeneDirex 100 bp DNA Ladder RTU, FroggaBio Inc., Toronto, ON, Canada) after electrophoresis at 90 V for 35 min. Gels were stained with ethidium bromide and images were captured using a Gel Doc™ XR+ System (Bio-Rad Laboratories Ltd., Mississauga, ON, Canada).

PCR fragments from each reaction were extracted from agarose gels and purified using the QIAquick Gel Extraction kit (Qiagen) following the manufacturer’s protocol. PCR product sample concentration was verified using a NanoDrop ND-1000 UV-Vis Spectrophotometer (Thermo Fisher Scientific, Ottawa, ON, Canada) and were sent to The Centre for Applied Genomics in Toronto, ON, Canada for sequencing. Genetic and predicted amino acid sequences from zebra finches and hummingbirds were aligned with each other and with published sequences for mouse, and chicken using CLUSTALW [34]. Sequence identity and similarity for each GLUT were then determined using standard program outputs.

### Immunoblots

GLUT1 and 4 protein expression was examined in the zebra finch pectoralis, heart, liver, brain, intestine, kidney, and ankle-extensor muscle group (e.g. gastrocnemius, soleus). Protein expression was examined in the hummingbird pectoralis, heart, liver, and brain. Some tissues used in RT-PCR analysis (or examined in the zebra finch, but not the hummingbird) were not examined for protein expression due to insufficient tissue sample mass. With 3 exceptions, expression was examined in a minimum of 3 samples from 3–9 individual birds of each species, depending on tissue, for both GLUT1 and GLUT4. GLUT1 expression in zebra finch intestine and GLUT4 expression in zebra finch intestine and kidney were examined in 2 samples, due to limited tissue mass. Samples from mouse skeletal and heart muscle were included as positive controls for GLUT1 and GLUT4 blots, respectively. Homogenization and blotting procedures followed those described in Sweazea and Braun (2006).

Approximately 50 mg of each tissue was homogenized in eight volumes of Krebs-Henseleit buffer at 4°C using either a disposable motor-driven pestle (VWR, Mississauga, ON, Canada) for soft tissues or a VDI 25 Adaptable Homogenizer (VWR) for muscle tissues. 40 µg of protein was loaded in wells for each lane of a 10% acrylamide gel and was run at 100 V for 120 minutes. BenchMark™ Pre-stained Protein Ladder (Invitrogen Technology: Carlsbad, California, USA) or Precision Plus Protein™ Dual Color Standards Ladder (Bio-Rad Laboratories Ltd.) was used to identify protein molecular weights as it displayed bands between 37 kDa to 64 kDa or 37 kDa to 75 kDa, respectively, near the expected weights of GLUT1 (≈65 kDa) [35] and GLUT4 (≈45–55 kDa) [36], [37]. Immunoblotting was not attempted for GLUT2 or GLUT3 because no commercial antibody showed sufficiently high homology to consensus amino acid sequences in the zebra finch or chicken (zebra finch GLUT2: XP_002193809.1; chicken GLUT3: NP_990842.1). Each blot was then transferred to a nitrocellulose membrane by electrophoresis at 90 V for 60 minutes. After blocking with 5% milk in TBST overnight, the membrane was incubated for 90 minutes in the primary antibody at room temperature: GLUT1 (1∶500; sc-7903, Santa Cruz Biotechnologies), or GLUT4 (1∶500; sc-1608, Santa Cruz Biotechnologies). After four 15 minute washes in TBST, the membranes were incubated for 1 hour in an HRP-conjugated goat anti -rabbit secondary antibody for GLUT1 (1∶5000; sc-2004, Santa Cruz Biotechnologies), and donkey anti-goat for GLUT4 (1∶5000; sc-2020, Santa Cruz Biotechnologies) in 2% milk and TBST. Membranes were then incubated in enhanced chemiluminescent reagent according to manufacturer protocol (Pierce ECL Western Blotting Substrate, Pierce Biotechnology: Rockford, Illinois, USA). Finally, the membranes were visualized using a Gel Doc™ XR+ System.

GLUT1 stained membranes were then stripped with 0.1 M glycine:HCl (pH 2.8) for 30 minutes at room temperature. Membranes were then washed with TBST and blocked for 1 hour with 5% BSA in PBS at room temperature. The membrane was then incubated with HRP-conjugated GAPDH Ab (1∶5000; ab9482, Abcam Inc, Cambridge, MA, USA) overnight at 4°C. Following incubation, the membrane was washed three times in PBS and imaged using the Gel Doc™ XR+ System, as above.

### Immunohistochemistry

Freshly dissected samples of zebra finch pectoralis and hummingbird pectoralis and heart (N = 4 or 5; each species) were coated in VWR Premium Frozen Section Compound (VWR International, Mississauga, Ontario, Canada), and frozen in 2-methylbutane cooled to −160°C by liquid nitrogen. Samples of hummingbird liver (N = 4) and brain (N = 2) were dissected and fixed in 30% sucrose/4% formalin for 7 days at 4°C before being coated in VWR Premium Frozen Section Compound and flash frozen as above. Samples were also obtained, and identically prepared, from mouse soleus, heart, brain and liver.

12 µm thick sections from these tissues were cut in a cryostat maintained at −24 to −20°C. Four to six sections were picked up on each microscope slide (Superfrost® Plus, Fisher Scientific, Ottawa, Ontario, Canada), with 18–54 serial sections obtained from each tissue. Slides were air dried at room temperature for 1–2 hours and then immediately stained.

Serial sections were stained to visualize either GLUT1 or GLUT4 using the same primary antibodies as in the immunoblots. GLUT staining began with acetone fixation and permeabilization for 5 min at 4°C. Subsequently, sections were washed twice in PBS (0.02 M sodium phosphate buffer, 0.15 M NaCl, pH 7.2) diluted with ddH_2_O. Sections were blocked in PBS containing goat serum (5%), bovine serum albumin (BSA; 10%) in PBS, and EDTA (5 mM) for 30 min. Slides were incubated overnight at 4°C with one of the two primary GLUT antibodies diluted 1∶200 in PBS. Following overnight incubation, samples were washed in PBS. Presence of the primary antibody was visualized by incubating sections for 30 min in the dark at room temperature with a donkey anti-rabbit IgG secondary antibody conjugated to an Alexa-488 fluorophore (Alexa Fluor®, NY, USA) at a 1∶300 dilution in PBS. Sections were washed in PBS prior to staining with DAPI (1∶100 dilution) for 15 min in the dark at room temperature to visualize nuclei. Sections were washed again in PBS and fixed in 4% formalin in PBS. Negative controls were made by excluding primary antibodies from the staining procedure. Additionally, sections from hummingbird and finch pectoralis, as well as mouse soleus, were stained to visualize capillaries using the periodic acid-Schiff (PAS) stain modified from the technique described in Anderson (1975) [38] or PAS staining was carried out using the Sigma-Aldrich PAS kit (Sigma-Aldrich, St. Louis, MO, USA). Sections were fixed for 10 min in a modified Carnoy’s solution (80% ethanol, 15% chloroform, and 5% glacial acetic acid) at room temperature, followed by subsequent washes with double distilled H_2_O (ddH_2_O). Then, sections were incubated in 1% amylase for 25 min at room temperature, followed by ddH_2_O washes. Tissues were stained in 1% Schiff’s reagent for 5–20 min at room temperature, depending on tissue type. Staining was developed following 10 min of ddH_2_O washes. Subsequent dehydration and clearing was carried out at 2 minute intervals using 80% ethanol, 90% ethanol, 100% ethanol, 100% ethanol, and xylene sequentially. All sections were mounted in Dako fluorescent medium (Dako Canada Inc., Burlington, Ontario, Canada).

PAS-stained sections were visualized with a Zeiss Axioplan-2 imaging Light Microscope (Carl Zeiss Canada Ltd., ON, Canada) while fluorescently-stained sections were visualized on a Quorum WaveFX Spinning disk confocal microscope (Quorum Technologies Inc. Guelph, Ontario, Canada) using Volocity 3D image analysis software (PerkinElmer, Woodbridge, Ontario, Canada).

## Results

### RT-PCR

**Figure 1:**
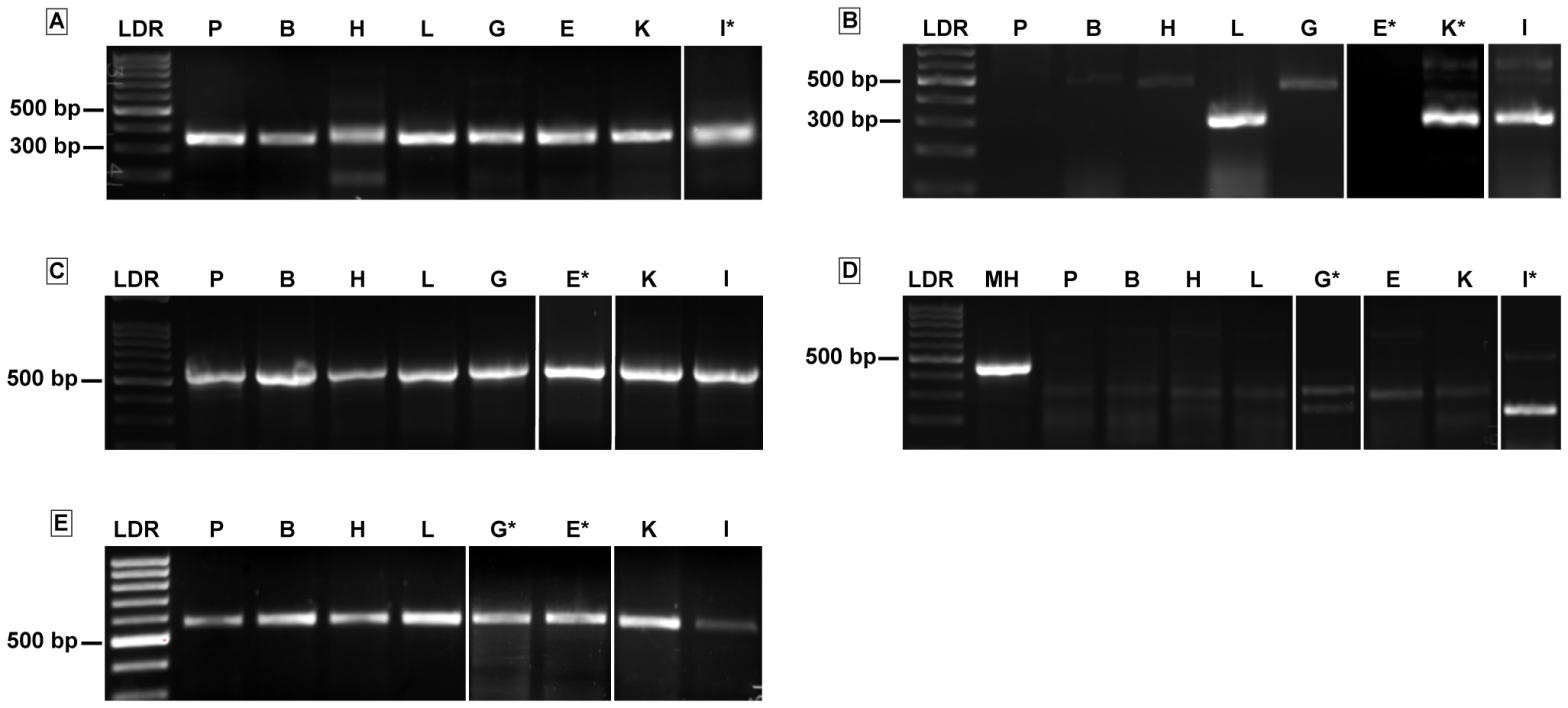
Glucose transporter mRNA expression in hummingbird tissues. Agarose gels (1.5%) of RT-PCR products for a) GLUT1 (340 bp), b) GLUT2 (305 bp), c) GLUT3 (543 bp), and d) GLUT4 (449 bp, expected product size from mouse), and e) GAPDH (585 bp). A 100 bp ladder was run in lane 1 of each gel. PCR reactions were performed on cDNA from hummingbird pectoralis (P), brain (B), heart (H), liver (L), ankle-extensor group muscles (G; e.g. gastrocnemius and soleus), wrist-extensor group muscle (E; e.g. extensor digitorum longus), kidney (K), and intestine (I), as well as cDNA from mouse cardiac tissue (MH; GLUT4 gel only) and samples of the reaction mixture were run in other lanes. Identical patterns of expression were observed using samples isolated from tissues of zebra finches (data not shown). Due to insufficient numbers of lanes per gel or because small tissue masses necessitated pooling of samples from 2 individuals, reaction products from some samples had to be run on separate gels. These are indicated by breaks in the image and by asterisks next to the lane headings (e.g. E*).

GAPDH gene expression in both zebra finches and ruby-throated hummingbirds was detected in each tissue examined appearing as a distinct band at the predicted size (585 bp; Fig. 1E). GLUT1 and GLUT3 gene expression were also detected in every tissue examined in both species (Fig. 1A, C). GLUT2 expression was detected in hummingbird and zebra finch liver, kidney, intestine, and in zebra finch pancreas. GLUT4 expression was confirmed in mouse heart (Fig. 1D), a tissue known to abundantly express this transporter [39]–[41]. However, no PCR products near the product size seen in the positive control (mouse heart) were observed in any of the avian tissues examined (Fig. 1D). In some instances, distinct bands of significantly smaller size were observed (e.g. intestine in Fig. 1D). However, subsequent sequencing of these products revealed no homology to any known GLUT sequences.

RT-PCR products from both hummingbird and zebra finch tissues were isolated and sequenced. Hummingbird GLUT1, 2, and 3 product sequences have been deposited in GenBank (Accession #KF492985, #KF492986, and #KF492987, respectively). Recovered bands from zebra finch tissues exhibited ≥99% homology with corresponding putative GLUT sequences published in GenBank. Thus, the more complete zebra finch GLUT sequences available on GenBank were used for further analysis of sequence homology among species. The three sets of sequenced hummingbird and zebra finch PCR products corresponded with regions encoding transmembrane segments 3–5 in human GLUT1, transmembrane segments 10 and 11 in human GLUT2, and transmembrane segments 4–6, part of transmembrane segment 3, through transmembrane segment 6 and approximately halfway through the subsequent large intracellular loop in human GLUT3, respectively. Sequences determined from RT-PCR products recovered from ruby-throated hummingbird tissue exhibited variable homology with published zebra finch sequences. Specifically, GLUT1 hummingbird and zebra finch sequences shared 99% identity. GLUT2 and GLUT3 sequences exhibited 85 and 88% identity, respectively. Patterns of sequence identity among hummingbird or zebra finch GLUTs and those from chicken or mouse were similarly variable, with GLUT1 showing the greatest sequence conservation while GLUT2 and GLUT3 showed variable, but consistently lower, identity. These data are summarized in Table 2.

**Table 2:** GLUT cDNA sequence identities.

		DNA sequence identity (%)		
		Zebra finch	Chicken	Mouse
**GLUT1**				
	Ruby-throatedhummingbird	99	89	79
	Zebra finch	–	89	82
**GLUT2**				
	Ruby-throatedhummingbird	85	83	72
	Zebra finch	–	84	71
**GLUT3**				
	Ruby-throatedhummingbird	88	88	71
	Zebra finch	–	85	67

Sequences for ruby-throated hummingbird are based on partial gene products of RT-PCR reactions. Partial sequences based on zebra finch RT-PCR products showed >99% identity with published sequences in GenBank. Thus, sequences from zebra finches and other species listed used for comparison are taken from GenBank.

### Immunoblots

**Figure 2:**
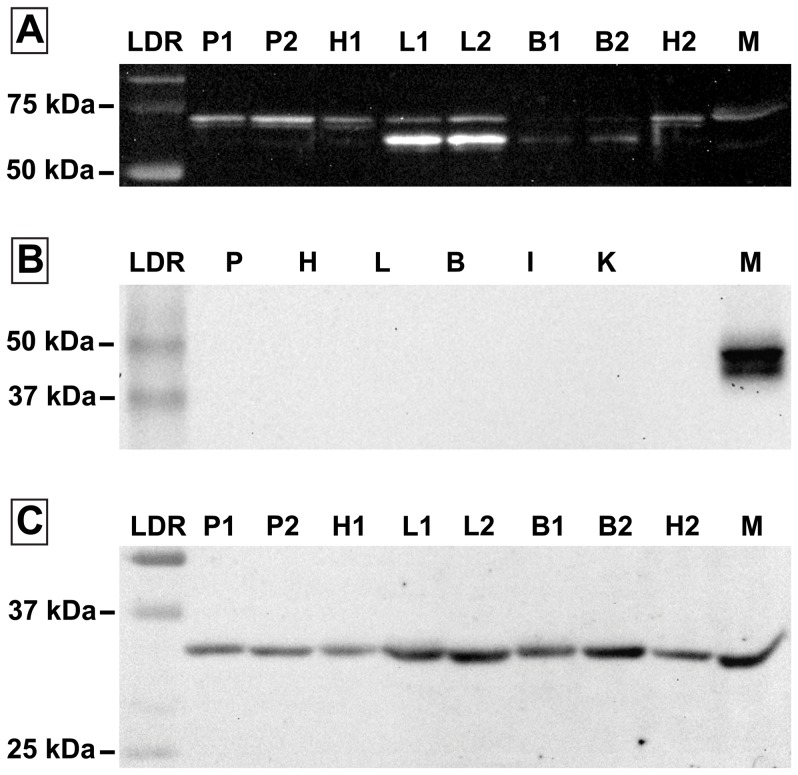
Glucose transporter protein expression in hummingbird tissues. Western blots using primary antibodies against a) GLUT1, b) GLUT4, and c) GAPDH. Samples were included from hummingbird pectoralis (P), brain (B), heart (H), liver (L), and for GLUT4 only, intestine (I) and kidney (K). Blots for GLU1 (a) and GAPDH (c) include samples from two different individual hummingbirds (e.g. P1 and P2). Samples from mouse (M) soleus (a, c) and cardiac (b) tissue are included as positive controls.

Western blot analysis confirmed the presence of a 67 kDa protein band (near the consensus molecular weight for GLUT1) in the mouse skeletal muscle and each of the avian tissues examined using the GLUT1 antibody (Fig. 2A). Interestingly, a distinct doublet pattern was observed in hummingbird liver and brain tissue samples probed with the GLUT1 antibody. In these cases, the second band was approximately 55 kDa and was typically more intense than the expected higher molecular weight band. Using the GLUT4 antibody, a protein with the expected molecular weight of 55 kDa was detected in the mouse heart (Fig. 2B). No protein bands were detected in any avian tissue using the GLUT4 antibody (Fig. 2B). GAPDH protein was successfully detected in all tissues examined, appearing near the expected molecular weight of 37 kDa (Fig. 2C).

Using the sequence data obtained as described above, we generated predicted partial amino acid sequences for hummingbird GLUT1, 2, and 3 proteins. Our sequences did not include the region containing a putative start codon. As a result, we assumed the predicted sequence which showed highest identity to other published GLUT sequences was that which would have correlated with the alignment of an identifiable start codon and used this predicted sequence for further analysis. All other GLUT amino acid sequences (including those from zebra finch) were obtained from GenBank. Variation in amino acid sequence identity among species followed patterns observed among genetic sequences. Ruby-throated hummingbird and zebra finch GLUT1, GLUT2, and GLUT3 amino acid sequences exhibited 92%, 84% and 86% identity, respectively. Amino acid sequence similarity between the two species was 96%, 88%, and 93%, for GLUT1, GLUT2, and GLUT3, respectively. Sequence identity/homology among taxa are listed in table 3.

**Table 3:** GLUT amino acid sequence identities and similarities.

		Amino acid sequence identity (similarity) (%)		
		Zebra finch	Chicken	Mouse
**GLUT1**				
	Ruby-throated hummingbird	98 (98)	96 (97)	86 (90)
	Zebra finch	–	98 (99)	88 (95)
**GLUT2**				
	Ruby-throated hummingbird	84 (88)	84 (88)	62 (84)
	Zebra finch	–	86 (93)	61 (81)
**GLUT3**				
	Ruby-throated hummingbird	90 (95)	90 (95)	73 (88)
	Zebra finch	–	87 (93)	70 (84)

Sequence identity and, in parentheses, similarity are listed for each paired comparison. Sequences for ruby-throated hummingbirds are extrapolated from an optimal alignment based on RT-PCR products of the partial cDNA sequence (see text). Sequences from all other species are those published in GendBank.

### Immunohistochemistry

**Figure 3:**
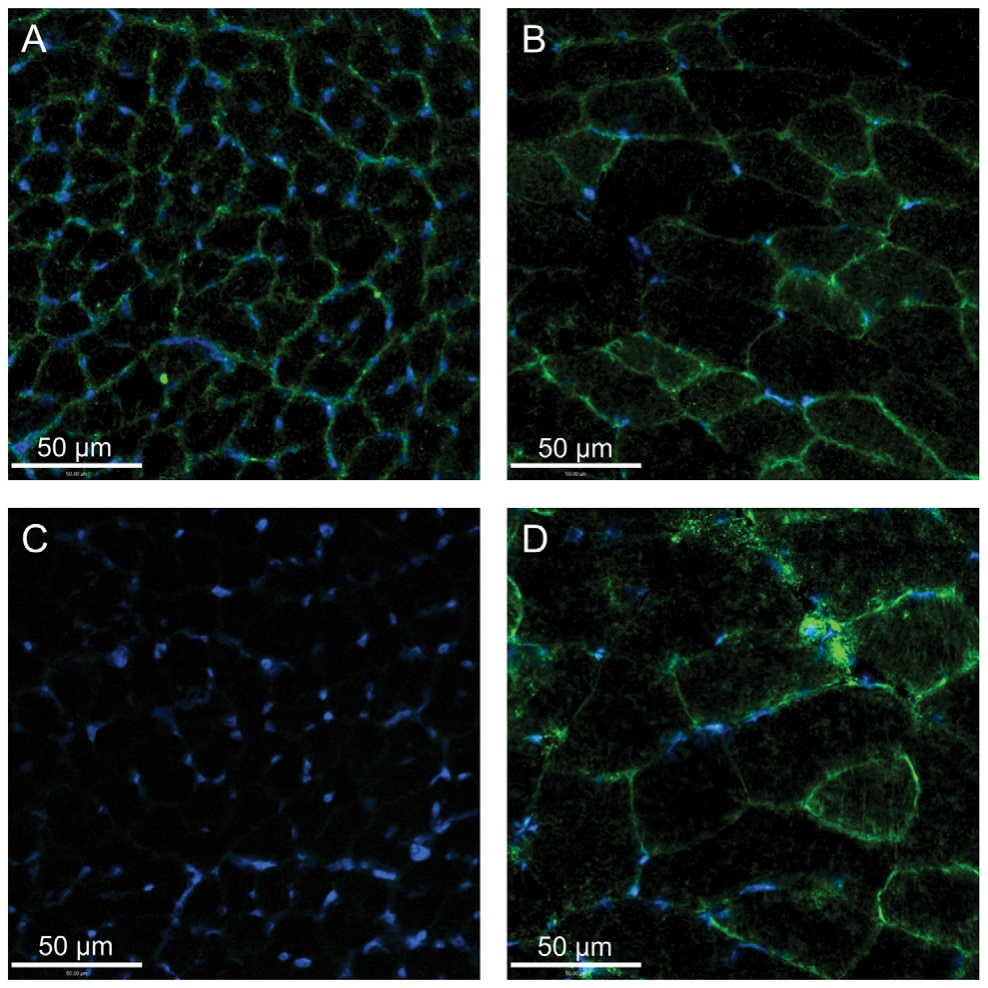
Glucose transporter staining in hummingbird and mouse skeletal muscle. Immunohistochemically-stained cross-sections of hummingbird pectoralis (a, c) and mouse gastrocnemius (b, d) muscle. Panels a and b) Immunostaining of tissues with GLUT1 primary antibody, visualized with a FITC-conjugated secondary antibody (green). Panels c and d) Immunostaining of tissues with GLUT4 primary antibody, visualized with a FITC-conjugated secondary antibody (green). Note, GLUT1 staining of the hummingbird pectoralis (a) is homogenous, and fiber sizes are all similar, reflecting the homogeneity of fiber type (type IIa; Fast oxidative-glycolytic). GLUT1 (and GLUT4) staining in the mouse gastrocnemius (b, d) is heteogenous and fiber diameters are varied, reflecting the diverse fiber type makeup of this muscle. Hummingbird pectoralis exhibited no staining using the GLUT4 antibody (intensity similar to use of secondary antibody alone; data not shown). Tissues in each panel were counterstained with DAPI in order to visualize nuclei (blue).

**Figure 4:**
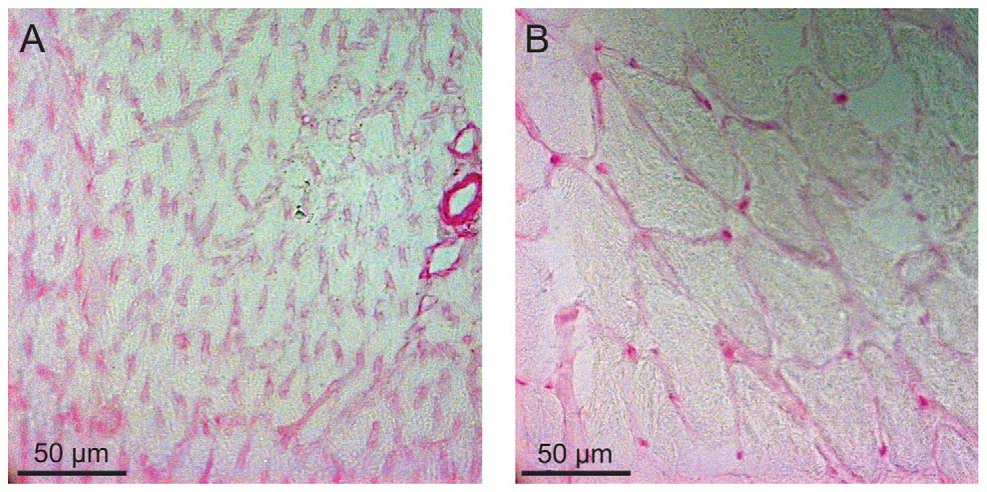
Visualization of capillaries in hummingbird and mouse skeletal muscle. Cross-sections of hummingbird pectoralis (a) and mouse gastrocnemius (b) muscle subjected to Periodic Acid-Schiff staining to visualized capillaries. Staining was much more intense in the hummingbird tissue reflecting the relatively greater capillary density in this tissue.

**Figure 5:**
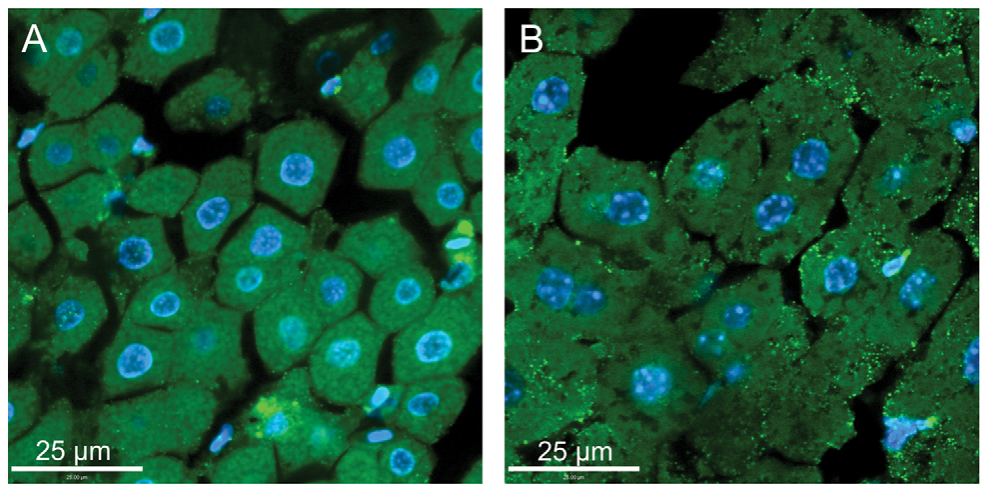
Glucose transporter staining in hummingbird and mouse liver. Sections of hummingbird (a) and mouse (b) liver tissue stained with GLUT1 primary antibody. Staining was visualized with a FITC-conjugated secondary antibody (green). Sections were counterstained with DAPI to visualize nuclei (blue).

**Figure 6:**
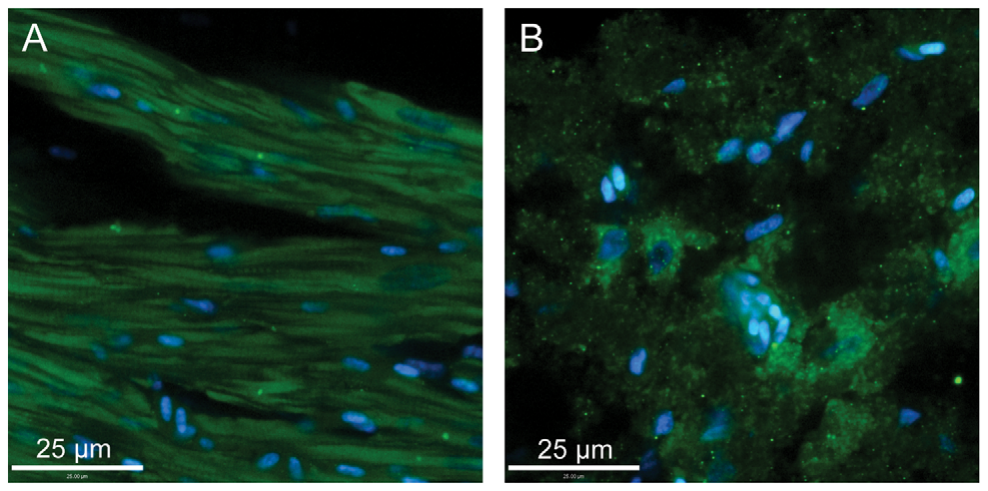
Glucose transporter staining in hummingbird heart and brain. Sections of hummingbird (a) heart and (b) brain tissue stained with GLUT1 primary antibody. Staining was visualized with a FITC-conjugated secondary antibody (green). Sections were counterstained with DAPI to visualize nuclei (blue).

Immunostaining patterns for GLUT1 were similar between hummingbird and zebra finch pectoralis. Staining was most intense along pectoralis fiber membranes (Fig. 3A), colocalizing with intense PAS staining (Fig. 4), suggesting GLUT1 was expressed in association with capillaries. This pattern was similar to that seen in mouse skeletal muscle except that staining of mouse gastrocnemius muscle was heterogenous (Fig. 3B). GLUT1 staining in other tissues was distinct. GLUT1 staining appeared homogenously distributed throughout both in avian and mouse liver cells (Fig. 5). GLUT1 staining was detected in both avian and mouse heart tissue, appearing homogenously distributed (Fig. 6). In addition, GLUT1 was detected in both avian and mouse brain tissue, though, within tissues, staining was more intense in some cells compared to others (Fig. 6).

GLUT4 staining was also seen in the mouse gastrocnemius muscle tissue, both along cell membranes and in some cases within cells (Fig. 3D), indicative of GLUT4 presence on intracellular vesicles, as has been extensively reported [42], [43]. In contrast, we failed to detect GLUT4 protein in any avian tissues. Avian tissues incubated with GLUT4 antibody stained with very low intensities similar to the levels observed in negative controls (incubation of tissues with secondary antibodies only; e.g. Fig. 3C).

## Discussion

Overall, patterns of GLUT transporter expression at the transcript (GLUT1, 2, and 3) and protein (GLUT1) level were similar to those reported in mammals and other birds [10], [19], [33], [40]. The exception is GLUT4, for which we failed to detect either mRNA or protein that showed any similarity to mammalian sequences in either hummingbirds or zebra finches. The apparent lack of a GLUT4 in either hummingbird or zebra finch skeletal muscle or cardiac tissue is in contrast to results from mammals [10], [40], [44] and, at the genetic level at least, some teleost fish [45], [46]. It is, however, consistent with the growing body of data from other avian taxa [19]. A search of the chicken and zebra finch genomes (which are completely or nearly completed described) reveals no sequence with substantial similarity to any known GLUT4 sequence. Thus, it seems increasingly clear that at least a few diverse avian taxa, and possibly the whole group, may lack a functional GLUT4 homolog.

Blood glucose levels in fasted hummingbirds are among the highest reported among vertebrates and more than double after feeding on nectar [25]. Hummingbirds forage for nectar frequently and continuously throughout the day, passing nectar from their crop to the rest of the digestive system in a similarly rapid and regular manner [47]. As a result of this relatively continuous flux of nectar through the digestive system, and thus into circulation, their blood sugar levels likely remain consistently high throughout the foraging period. The lack of an insulin-responsive GLUT4-like protein in hummingbirds may be a contributing factor to both the observed fasting (≈14 mM), and fed hyperglycemia (≈40 mM) in this species [25], as has been proposed for other avian taxa [19], [33]. While birds generally seem insulin insensitive, the efficacy of this hormone in regulating hummingbird blood glucose is unknown. Still, based on available evidence in other birds, and on the lack of a GLUT4 protein, we hypothesize that hummingbirds would be similarly insulin insensitive.

Fasted hummingbirds fuel perching and hovering flight almost exclusively with fats, and switch rapidly and completely to fueling metabolism with newly ingested carbohydrates as blood sugar levels rise in the minutes after feeding [2]–[4], [48]. This ability to switch between fuel type and source based solely on dietary status is unusual among vertebrates. Both the type and sources of fuels oxidized during exercise are linked to exercise intensity in most mammals. At low exercise intensities, lipids derived from intramuscular and adipose tissue stores provide most of the chemical energy used. As exercise intensity increases, most mammals display a progressive shift towards greater reliance on both intramuscular and circulating carbohydrate [49], [50]. In contrast, muscle fuel use is uncoupled from exercise intensity in hummingbirds, as they can fuel energetically expensive hovering flight equally well with fats (when fasted) and with dietary sugars (when fed). The coupling of exercise intensity and fuel use in mammals is explained, in part, by the translocation of a distinct pool of GLUT4 in response to increased contractile activity [17], [18]. The lack of a GLUT4, and thus enhancement of sugar uptake by muscles in response to increased activity, in hummingbirds is consistent with the uncoupling of exercise intensity and fuel use.

Blood glucose levels in fasted hummingbirds are considerably lower than in fed hummingbirds, though they are, in comparison to almost all other vertebrates, still high (≈14 mM) [25]. This implies that, during periods of fasting, rates of sugar uptake by metabolically active tissues (e.g. muscle and liver) are depressed. This change in sugar uptake rate may be due to relatively low affinity of GLUTs for glucose or fructose, such that only the comparatively high blood sugar concentrations observed in fed birds (≈30–40 mM) lead to high rates of uptake. However, variation in the rate of sugar transport into tissues among fasted and fed states could also be the result of post-translational regulation of function or variation in subcellular localization of the GLUTs that are present. This study was not designed to examine variation in GLUT function or localization in relation to feeding status, and all birds used in this study were considered well-fed up to the time of sacrifice (see Methods section). Ongoing research will examine such potential variation in hummingbird GLUT function or tissue localization.GLUT1 staining in the hummingbird pectoralis was relatively homogenous, localized primarily to fiber membranes (Fig. 3A), and capillaries, based on overlap with PAS staining (data not shown). In addition, GLUT1 staining was detected in hummingbird and zebra finch erythrocytes that were captured in a few larger vessels present in isolated muscle sections (data not shown). The strong staining of avian erythrocytes for GLUT1 mirrors such staining observed in mammalian erythrocytes [9], [10], [40]. Staining was similar in the zebra finch pectoralis. By comparison, GLUT1 (and GLUT4) staining in the mouse gastrocnemius muscle was heterogenous, with some fibers staining more intensely than others (Fig. 3B, D). Density of GLUT transporters, and thus intensity of staining in mouse skeletal muscle differs among distinct fiber types. In contrast, the flight muscles of hummingbirds, zebra finches, like many other small-bodied, volant avian species, are composed of a single fiber type (type IIa, fast-twitch oxidative glycolytic) [51], [52]. Homogenous staining of these muscles suggests that, like distribution of myosin heavy chain isoforms and mitochondrial density, capacities for uptake of sugars from circulation (at least via GLUT1) are also homogenous. Given that GLUT1 staining intensity differs among fiber types in mammalian skeletal muscle, we hypothesize that patterns of GLUT1 staining among fibers in the hummingbird and zebra finch ankle-extensor muscle group (e.g. gastrocnemius, soleus) would also be heterogenous since these muscles, unlike the flight muscles, are composed of multiple fiber types [52]. Because ankle-extensor muscle groups in each species are so small, and because these tissues were prioritized for RT-PCR and immunoblot use, we were unable to conduct immunostaining studies to test this hypothesis.

Genetic and amino acid sequence similarity among species was highest for GLUT1 and, on average, progressively lower for GLUT2 and 3. Generally, sequence similarity is quite high for GLUT1 among mammals (>97%) and it seems this trend holds among birds as well, with hummingbird GLUT1 showing >90% amino acid sequence homology to zebra finch and chicken GLUT1 [53]. The lower similarity of GLUT2 and 3 sequences among avian and mammalian taxa is consistent with fact that antibodies developed against these proteins in mammals are poorly cross-reactive with their avian homologs.

In hummingbirds, much of the glucose (and, presumably, other hexoses) is absorbed across the gut wall via a non-mediated, paracellular pathway [8], [54]. Reliance on paracellular absorption of sugars in the gut facilitates high rates of flux of sugars independent of GLUT transporter function, an adaptation which has permitted the evolution of smaller, lighter mass digestive machinery in hummingbirds and other small, volant vertebrates [54]. Still, it appears that the *V*_max_ for cellular-mediated (i.e. involving GLUT transporters) glucose transport across the hummingbird gut wall is comparatively large [8]. That said, passive diffusion of into cells (e.g. liver, muscle, brain) does not occur. Unlike in the hummingbird gut, the surface area (normalized to body mass, or muscle volume, respectively) across which sugars move from circulation into muscle fibers is much greater in hummingbirds than in most other vertebrates [55]. It is likely that, just as with the diffusion of oxygen, small muscle fiber cross-sectional area (high fiber surface area to volume ratio) and high capillary density facilitate high rates of sugar transport per volume of tissue facilitated solely by GLUT transporters [5], [55], [56]. Still, because the functional capacities of hummingbird GLUTs are undescribed it remains only an assumption that GLUT mediated transport is sufficient to explain observed high rates of sugar uptake into active flight or liver tissues.

A recent study shows that the kinetics of oxidation of ingested fructose in hovering hummingbirds is at least as rapid as that for ingested glucose [2]. This raises the possibility that these animals, which receive ∼50% of their calories in the form of fructose [57], [58], may be capable of transporting this hexose directly from circulation into their muscle cells and oxidizing it at rates much higher than maximal capacities seen in most mammals [59], [60]. In mammals, only a few of the 14 GLUT transporters are believed to have significant affinity for fructose. These are GLUT2 (class 1), GLUT5, 11 (class 2), and GLUT8 (class 3) [10]. While we detected GLUT2 gene expression in the liver, it was not detected in the skeletal muscle of either bird. Other studies have failed to find GLUT2 expression in avian skeletal muscles [61]. Thus, it seems highly unlikely that GLUT2 protein is present in the hummingbird flight muscles where it could provide some capacity for the uptake of fructose. We did not examine expression patterns of the other three fructose transporters. The only one of these transporters localized to skeletal muscle in mammals is GLUT11 [10]. Patterns of GLUT5 and 11 expression in avian tissues are not known. GLUT8 cDNA has been detected in chicken pectoralis, though at much lower levels than in other tissues [61], [62]. GLUT8 protein expression patterns in birds remain undescribed. In general, affinities and functional capacities of any avian GLUTs for fructose are not known. The regions of GLUT1-3 sequenced as part of this study generally fall outside the specific regions that have, in humans, been shown to be especially variable among GLUT family members (e.g. either terminal region or extracellular loops 1 or 9 [36]) or in which specific sequence motifs have been associated with functional variation (e.g. the QLS motif present in transmembrane segment 7 [63]). Further study is warranted. For the time being, it remains unclear by what mechanisms significant rates of fructose transport to and from metabolically active tissues such as flight muscle and liver may be enabled in hummingbirds or any other avian taxa.

In summary, we show that hummingbirds and zebra finches do not express a GLUT4 transporter. The consensus emerging from a growing body of work on avian taxa [19], [33], [62], [64] is that this group generally does not possess a functional GLUT4 protein and that the gene may have been lost early in its evolution. We also show that expression patterns for GLUT1, 2 and 3 are consistent with reports from both birds and mammals [10], [19], [40].

The lack of a GLUT4 protein in hummingbirds may be related in this group, as it is hypothesized to be in other avian taxa [19], [33], to persistent hyperglycemia during both fasting and fed periods. Further, we conclude that the lack of a contraction-responsive GLUT4 in hummingbirds at least partly explains an uncoupling between exercise intensity (e.g. hovering flight) and muscle substrate oxidation that is otherwise consistently seen in most mammals [49], [50].

We highlight the need for continuing work to better understand the function and regulatory role of glucose transporters in the movement of sugars between the circulatory system and active tissues in hummingbirds and other avian taxa. Hummingbirds demonstrate a remarkable capacity for the rapid flux of sugars from the intestine, through the circulatory system, and to active tissues, such as flight muscle and liver [2]–[4]. Yet, qualitative differences in class I GLUT expression which might explain such capacities are not evident. Further work is needed to discover if GLUT transporter kinetics, and not simply transporter density or abundance, are substantially enhanced in hummingbirds and other energetic avian taxa.
